# Prophylactic Effects of Lidocaine or Beclomethasone Spray on Post-Operative Sore Throat and Cough after Orotracheal Intubation

**Published:** 2015-05

**Authors:** Nadia Banihashem, Ebrahim Alijanpour, Bahman Hasannasab, Ali Zarei

**Affiliations:** 1 *Department of Anesthesiology, Babol University of Medical Sciences, Babol, Iran. *

**Keywords:** Beclomethasone, Lidocaine, Sore throat

## Abstract

**Introduction:**

Post-operative sore throat and cough are common complications of endotracheal intubation. These conditions may be very distressing for the patient and may lead to unpleasant memories. This study was performed in order to determine whether beclomethasone and lidocaine spray could reduce the frequency of post-operative sore throat and hoarseness after tracheal extubation.

**Materials and Methods:**

Ninety women (18–60 years of age) with an American Society of Anesthesiologists (ASA) physical status I or II and undergoing elective mastoidectomy were randomized into three groups of 30 patients. The endotracheal tubes in each group were sprayed with 50% beclomethasone, 10% lidocaine hydrochloride, or normal saline (control group) before endotracheal intubation. Patients were examined for sore throat (none, mild, moderate, or severe), cough, and hoarseness at 1 and 24 h after extubation.

**Results:**

There was a significantly lower incidence and severity of post-operative sore throat in the beclomethasone group than the lidocaine and control groups (P<0.05) at each observation time point. At 24 h after extubation, the incidence and severity of sore throat and cough was significantly lower in the lidocaine compared with the control group. The incidence of hoarseness was not significantly different among the three groups.

**Conclusion:**

Spraying beclomethasone and lidocaine on the endotracheal tube is a simple and effective method to reduce the incidence and severity of post-operative sore throat.

## Introduction

Post-operative sore throat, cough, and hoarseness of voice are common complications after general anesthesia, with an incidence varying from 40–100%. These conditions are an uncomfortable and distressing sequela after tracheal intubation (1,2). Inflammation may be important in the pathogenesis of post-operative sore throat (3). Tracheal intubation can result in trauma and nerve damage, which may also account for post-operative throat symptoms such as hoarseness and dysphagia (4,5). 

Various methods have been used to attenuate the sore throat after general anesthesia, with variable success. Use of small-sized endotracheal tubes, careful orotracheal intubation, spraying the endotracheal tube cuff with lidocaine, intubation after full relaxation, gentle oropharyngeal suctioning, minimizing intra-cuff pressure, extubation when the tracheal tube cuff is fully deflated, and gargling with aspirin and ketamine have been reported to decrease the incidence of post-operative sore throat (1-6). Endotracheal spraying with lidocaine has been widely accepted as a useful method for obtunding the pressor response to intubation (7).

Two studies have shown that lidocaine decreases the incidence of cough and sore throat after general anesthesia (8,9). The prophylactic use of topical and systemic steroids was found to reduce the incidence of sore throat and cough during recovery, probably by modifying the inflammatory process caused by tissue injury (5,10). The aim of this study was to determine whether beclomethasone and lidocaine spray could reduce the frequency of post-operative sore throat and hoarseness after tracheal extubation.

## Materials and Methods

This randomized double-blind study was conducted after Institutional Ethics Committee approval and receipt of written informed consent. Ninety women (n= 30 per group) with an American Society of Anesthesiologists (ASA) physical status I–II, aged 18 to 50 years and scheduled for mastoidectomy under general anesthesia with orotracheal intubation were included in this study. Patients were randomized into one of three groups: lidocaine, become- thasone, or control.

Patients with a difficult airway, a history of recent respiratory tract infection, a requirement for a nasogastric tube, a history of perioperative sore throat, obesity, neurological or psychological disorders as well as drug abuse were excluded. In addition, patients in whom laryngoscopy was attempted more than once or in whom the duration of laryngoscopy was more than 15 s were excluded.

After standard monitoring and administration of 2 μg/kg fentanyl and 0.04 mg/kg midazolam, induction was accomplished with 5–6 mg/kg thiopental sodium and 0.5 mg/kg atracuriom. Endotracheal intubation was performed with high-volume, low-pressure cuffs (Supa Medical Devices, Tehran, Iran) using standard 3-Macintosh blades. All procedures were performed by a resident with at least 3 years of experience. The tracheal tubes were sprayed with 50% beclomethasone, 10% lidocaine, or normal saline before orotracheal intubation. All medications were sprayed by a nurse blinded to the treatment. The endotracheal tube (ET) cuffs were inflated with room air to a cuff pressure of 20–25 cm H2O. Cuff pressure was measured immediately after ET intubation and at the end of surgery using a manometer. 

The lungs were mechanically ventilated with N2O:O2 and isoflurane (0.7-1% end-tidal concentration) intraoperatively. Neuromuscular blockade was antagonized by neostigmine and atropine at the end of surgery. When patients opened their eyes and responded to verbal commands, the ET was removed after gentle suctioning of oral secretions. Patients were interviewed by a blinded investigator at 1 and 24 h after extubation for post-operative sore throat (POST), cough, and hoarseness. POST was assessed by a modified 4-point scale (0= no sore throat, 1= mild sore throat: complains of sore throat only on asking, 2 = moderate sore throat: complains of sore throat spontaneously, and 3 = severe sore throat: change of voice or hoarseness, associated with throat pain) (4). Other complaints, such as hoarseness, bucking, dysphonia, dysphagia, and nausea and vomiting were assessed as present or absent in the first 24 h after surgery.

The sample size calculation was performed on the basis of a previous study (11), in order to obtain at least 25–30% reduction in the incidence of sore throat, with α =0.05 and power 80%. A sample size of 25 patients was required for each group. Allowing for drop-out, we planned to study 30 patients in each group. 

Demographic data were analyzed using one way analysis of variance (ANOVA) for continuous variables and a chi-square test for categorical variables. The incidence of post-operative symptoms and side effects was analyzed using the Chi-square test and Fisher's exact test, whereas the severity of sore throat was analyzed using the Kruskal-Wallis and Mann-Whitney U tests.

## Results

All of the 90 patients enrolled completed the study. Patient characteristics were comparable across the three groups with respect to age, weight, intubation time, and mean arterial pressure and heart rate (Table. 1). 

The severity and incidence of sore throat was significantly lower in the beclomethasone and lidocaine groups compared with the control group (Table 2). The incidence of severe sore throat in the beclomethasone, lidocaine, and control groups was 0%, 10%, and 10% respectively (P= 0.2). Beclomethasone and lidocaine were effective in preventing cough in the first hour after surgery, but were ineffective at 24 h (Table. 2). The frequency of hoarseness of voice during the first 24 h post-operatively was comparable in the three groups. The frequencies of adverse events during the post-operative period were comparable among the groups (Table. 3). Local or systemic side effects were not observed.

**Table 1:** Patients characteristic and operative data

	**Beclomethasone** **(n = 30)**	**Lidocaine** **(n = 30)**	**Control** **(n = 30)**	**P-value**
Age (years)	43.70±10.73	41.87±7.71	42.10±13.35	0.77
Weight (kg)	75.13±7.46	71.4±9.11	71.10±8.17	0.11
Time of anesthesia (min)	118.50±30.29	106.33±27.09	105.87±32.04	0.18
Time of spontaneous breathing (min)	12.66±15.40	11.13±4.71	11.13±10.47	0.83
Mean arterial pressure (mmHg)	103.64±10.56	104.77±12.17	99.31±12.82	0.17
Heart rate (beat/min)	95.70±18.96	92.53±15.95	87.72±15.05	0.18

**Table 2:** Sore throat and cough data

		**Beclomethasone** **(n = 30)**	**Lidcaine** **(n = 30)**	**Control** **(n = 30)**	**P-value**
Severity of Sore throat	1 h after extubation	0.1/0 (0–1)*	0.5/0 (0–1)	0.6/0 (0–3)	0.04
	24 h after extubation	0.1/0 (0–1)*	0.4/0 (0–3)†	0.77/0 (0–3)	0.002
Incidence of sore throat	1 h after extubation	3 (10%)‡	7 (23%)	11 (36.7%)	0.05
	24 h after extubation	3 (10%)‡	7 (23%)†	15 (50%)	0.002
Cough	1 h after extubation	0 (0%)‡	5 (16.7%)	10 (33.3%)	0.002
	24 h after extubation	2 (6.7%)	6 (20%)	8 (26.7%)	0.11

Data are expressed as number of patients (%) or mean/median (range).

* P< 0.05, Beclomethasone group versus control group.

† P< 0.05, lidocaine group versus control group.

‡ P< 0.05, Beclomethasone group versus lidocaine group and control group.

**Table 3:** Spasm, bucking, nausea, vomiting, dysphagia and hoarseness data

	**Beclomethasone** **(n = 30)**	**Lidocaine** **(n = 30)**	**Control** **(n = 30)**	**P-value**
Spasm	1 (3.3%)	2 (6.7%)	3 (10%)	0.58
Bucking	0 (0%)	2 (6.7%)	3 (10%)	0.22
Nausea	8 (26.7%)	8 (26.7%)	9 (30%)	0.94
Vomiting	4 (13.3%)	6 (20%)	5 (16.7%)	0.78
Dysphagia	3 (10%)	5 (16.7%)	4 (13.3%)	0.74
Hoarseness	1 (3.3)	1 (3.3)	4 (13.3)	0.06

## Discussion

Our study shows that beclomethasone significantly decreased the incidence and severity of post-operative sore throat and cough compared with placebo. The incidence of hoarseness, bucking, spasm, or dysphagia was not significantly different among the groups in the present study. All patients in this trial were women and candidates for mastoidectomy. Therefore, gender was eliminated as a possible confounding factor.

This study confirms the relatively high incidence of sore throat, cough and hoarseness of voice after general anesthesia. The incidence of these outcomes ranged from 26.7–50%; consistent with the results of other studies (11-14).

We found that the incidence and intensity of sore throat in the beclomethasone group was lower than that in the control group. The effectiveness of a beclomethasone inhaler in the prevention of post-operative sore throat and cough has been reported previously in two studies (15,16). Tazeh-Kand et al. showed that inhaled fluticasone decreased the incidence of post-operative sore throat, cough, and hoarseness (11). Sumathi et al. found that the betamethasone gel applied over the tracheal tube effectively decreased post-operative sore throat, cough, and hoarseness of voice compared with lidocaine gel (17). Thomas et al. found that prophylactic intravenous dexamethasone reduced the incidence and severity of sore throat following endotracheal intubation (18). The causes of cough, hoarseness, and sore throat after laryngoscopy and orotracheal intubation are attributable to local irritation, inflammation, and edema in some parts of the pharynx, larynx, vocal cords, or trachea. It is thought that topical corticosteroids decrease irritation, inflammation, and edema in the upper airway as well as the incidence and severity of sore throat and cough (12,13). Stride et al. found that topical application of 1% hydrocortisone cream was ineffective in reducing the incidence of post-operative sore throat. These authors only applied topical hydrocortisone from the distal tip to 5 cm above the cuff (19).

This study showed that lidocaine was effective in decreasing the incidence sore throat, but not cough or hoarseness. A previous study showed that lidocaine spray was effective in reducing the incidence of cough after tracheal extubation in surgeries of less than 2-h duration (4). 

The precise mechanism of the suppression of cough and sore throat by lidocaine is not clearly known. Several studies have reported that local anesthetics significantly reduce inflammation and tissue damage. The anti-inflammatory mechanism of local anesthetics is related to the inhibition of ion exchange by the membrane channels themselves (20–22). Takekawa et al. observed that intravenous lidocaine decreases the incidence and severity of sore throat. The strong stimulation of intubation may excite sensory C–fibers (23). Lidocaine may reduce the release of neuropeptides, while direct central suppression can also occur (24). In a previous study, spraying 10% lidocaine on an orotracheal tube cuff increased the severity of sore throat (4). Ten percent lidocaine solution contains additives in the solvent which may irritate the tracheal mucosa, potentially causing tracheal mucosa damage and increased severity of POST (4). Honma et al. reported that lidocaine spray decreased the incidence of sore throat, but lidocaine jelly increased the incidence of sore throat (25). Possible explanations for the differences between studies include differences in the anesthetic and interview techniques.

The safety and dosage of beclomethasone applied to the tracheal tube requires further investigation, even though no adverse effects were reported in this study.

## Conclusion

Spraying beclomethasone and lidocaine on the endotracheal tube is a simple and effective method of reducing the incidence and severity of post-operative sore throat in patients under general anesthesia.
